# A review of hair product use on breast cancer risk in African American women

**DOI:** 10.1002/cam4.613

**Published:** 2016-01-15

**Authors:** Laura Stiel, Paris B. Adkins‐Jackson, Phyllis Clark, Eudora Mitchell, Susanne Montgomery

**Affiliations:** ^1^ Department of Social Work and Social EcologyLoma Linda UniversitySan BernardinoCalifornia; ^2^ Asante Wellness, LLCLos AngelesCalifornia; ^3^ Healthy HeritageRiversideCalifornia; ^4^ Quinn Community Outreach CorporationMoreno ValleyCalifornia; ^5^ School of Behavioral HealthLoma Linda UniversityLoma LindaCalifornia

**Keywords:** African Americans, breast neoplasms, hair, health status disparities, women's health

## Abstract

The incidence rate of breast cancer for African American women has recently converged with that of non‐Hispanic White women in the United States, although African Americans have a higher mortality rate due to this disease. Although most research exploring health disparities associated with this phenomenon has focused on differences between women based on biology and behavior, both the academic and lay communities have begun to explore the potential role of environmental exposure to estrogen and endocrine disrupting chemicals (EDCs). This study reviews the current state of the science associating one such means of exposure, hair products containing EDCs, with breast cancer risk in African American women. We found a growing body of evidence linking: (1) environmental estrogen and EDC exposures to breast cancer risk, (2) the presence of such chemicals in personal care products, including hair products, and (3) the use of certain hair products with potential breast cancer risk in African Americans. At the same time, there is also increasing concern in the lay community about this risk. These results indicate the need for additional research, and the opportunity to benefit from strategic partnerships in community‐collaborative approaches in order to better understand the potential “cost of beauty.”

## Introduction

Breast cancer is the most common cancer among African American women 1. While the incidence rate of breast cancer used to be lower for African American compared to White women of all ages, rates for the two groups have recently converged 2. In addition, the incidence rate is higher for African Americans ages 45 or younger 1. Further, mortality rates due to breast cancer are highest in African American women compared to any other group 1. Notably, the mortality rate for African Americans under age 50 is nearly twice that of other women in the same age range 3. Across all age groups, African Americans are more likely to be diagnosed with late‐stage breast cancer than Whites 4, 5.

While race is a social construct, the concept is sometimes conflated with biological differences between people, and it can be difficult to separate the two within the literature. Danforth argues that four breast cancer disparities between African Americans and Whites—early age of onset, more advanced stage of the disease at presentation, more aggressive histologic changes, and worse survival—are the result of biological properties influenced by nonbiological features 6. In his model, socioeconomic characteristics, healthcare access, and reproductive factors may promote, inhibit, or facilitate biological activities and differences, or act as confounding factors. However, this model, as with other traditional research, neglects the potential role of environmental influences, such as environmental exposure to estrogen or chemicals mimicking the effects of estrogen, through the air, water, food, or skin, which are factors that have increasing awareness in breast cancer risk research 7.

### Endocrine disrupting chemicals, personal care products, and breast cancer risk

Endocrine disrupting chemicals (EDCs) are chemicals, or chemical mixtures, that interfere with the normal activity of the endocrine system, which is made up of many tissues and hormones in the body that are responsible for human growth, development, and reproduction 8. EDCs can act directly on hormone receptors as mimics or antagonists, or on proteins that control hormone delivery 8. There are many known or suspected EDCs, but this review is focused on natural and synthetic EDCs that are exogenously applied to the scalp and hair through the use of personal care products. Studies exploring this issue have thus far considered EDCs classified as estrogens, phthalates, and parabens.

EDCs that mimic the effects of estrogen in the body may contribute to disease risk because exposure to estrogen, endogenously and exogenously, is associated with breast cancer 9, 10, and a woman's lifetime risk of developing the disease increases with greater duration and cumulative exposure 11. Further, women who are younger at menarche 10 are at increased risk of breast cancer, and exposure to EDCs may impact premature sexual development. Parabens have been found in human breast tumor tissue 12, even in women who reported never having used underarm cosmetics, and persisted when controlling for age, time spent breast‐feeding, tumor location, and tumor estrogen receptor content 13. Serum levels of mono‐ethyl phthalate and bisphenol A (BPA), a synthetic chemical that mimics the effects of estrogen, have been positively associated with mammographic breast density, a strong marker of breast cancer risk 14. Urinary concentrations of mono‐ethyl phthalate have been positively associated with breast cancer risk 15, as well as the number of personal care products used 16, 17, and the use of hair products, among other personal care products, has been significantly associated with urinary phthalate concentration 16, 18.

However, the specific mechanisms by which EDCs may affect breast cancer etiology are still under investigation. Normal mammary gland development is regulated in part by hormones and occurs in stages during gestation, puberty, and pregnancy. EDCs may alter mammary gland development in ways that increase the risk of breast cancer, as exposure to EDCs during development may cause chemical alterations that make tissue more susceptible to a second exposure of a carcinogen 19. Alternatively, EDCs may prolong puberty 20 or act directly as carcinogens by changing the rate of mammary gland development or the branching and epithelial structure 11, 19, which can increase the rate or duration of epigenetic changes that destabilize chromatin integrity or lead to aberrant gene expression 20. Some EDCs may also stimulate the proliferation of cancer cells and increase their migratory and invasive properties 21, 22, 23, 24. The three EDC groups discussed in this study, which cover a range of individual chemicals, are shown in Table 1 with their general uses in hair products and the mechanisms that have been proposed for the ways they may contribute to breast cancer risk.

**Table 1:** Use of reviewed EDCs in hair products and possible mechanisms associated with breast cancer risk

EDC group	Use in hair products	Possible mechanism
Estrogens	Promote hair growth 51	Epigenetic changes leading to predisposition to tumorigenesis, altered mammary gland development, and cell proliferation 20
Phthalates	Fragrance 52	Alter mammary gland development through epigenetic changes 53, promote cell growth, and increase migratory and invasive properties in breast cancer cells 23
Parabens	Preservative 54	Induce growth of breast epithelial cells 25, increase migratory and invasive properties of breast cancer cells 24

Both dose levels and timing of exposure affect the reaction of the mammary gland to EDCs. Although EDCs pose a risk throughout one's life span, individuals are especially vulnerable when exposed in utero, and even when the exposure to EDCs is removed, women can still be impacted over generations as they inherit a particular type of gene regulation. Arguably, this is what occurred when the daughters of some women who took one type of EDC 25, the synthetic estrogen diethylstilbestrol (DES), while pregnant developed uterine abnormalities and cancers as young adults 26. Studies have shown that women ages 40 and above who were exposed to DES in utero through their mothers have an increased risk of breast cancer due to their exposure 27, 28. Indeed, some researchers suggest this increased risk may also extend to third and fourth generations 29. Similarly, rodent studies involving fetal BPA administration indicate that prenatal exposure to this EDC may lead to increased risk of breast cancer 30.

As environmental estrogen and EDC exposures have been associated with breast cancer risk, and the presence of such chemicals are found in personal care products, including hair products, this review assesses associations between hair products known or suspected to contain such ingredients, and breast cancer risk in African American women.

## Methods

Peer‐reviewed studies were identified through searching EBSCO, PubMed, and Google Scholar using multiple combinations the following terms: African American/Black, breast cancer, hair, hair products, personal care products/PCPs, endocrine disrupting chemicals/EDCs/endocrine disruptors, and estrogen(s). The abstracts of search results were read for relevancy to the review; studies appearing to relate to the topic were then read and assessed for inclusion. To be included in the review, studies must have met the following criteria: (1) focus on African American girls or women; (2) consider exposure to hair products with known or potential hormonally active ingredients; and (3) engage in analyses related to breast cancer risk. Studies associated with breast cancer survivor behavior or breast cancer education were not included. References from articles found were also examined and considered for inclusion. Given that this is still a new area of research, abstracts found during the search were not excluded. In all, six articles and one abstract were included in the review, reflecting a small but growing body of research linking the use of certain hair products to breast cancer risk in African American women.

## Results

A number of different hair products were assessed in the studies reviewed, including hair preparation, relaxer, perm, cream, root stimulator, oil, lotion, and conditioner. These products are applied to the scalp and/or hair in different ways, are in contact with the scalp for different periods of time, contain different chemicals, and enter the body through different mechanisms. In addition, the use of some products, such as relaxers, can result in scalp lesions and burns, whereas the use of other products will not. These variations in exposure should be taken into account when considering the findings described below (summarized in Table 2).

The research exploring links between EDCs in personal care products and breast cancer risk is especially compelling when investigating health disparities, as hair products commonly used by African Americans often vary from those used by Whites. For example, White women rarely use hair relaxers, but one study found that 94% of African American women surveyed under age 45 and 89% aged 45 and above used them 31. According to one review 32, some studies have found that African Americans use more personal care products that contain hormones and placenta than Whites, and may be exposed to them as infants, toddlers, and in utero through their pregnant mothers. Further, the authors hypothesize that penetration and absorption of hair products are increased with the use of heat, which is regularly used in their application 32. This study identified ten reports of premature or abnormal sexual development related to use of hormone‐containing products, including hair products with estrogen. The review concludes that use of such products by African American girls and women helps explain the higher incidence of breast cancer in younger African Americans, and that the early and cumulative lifetime exposure to these products contributes to the increased lethality of the disease in this population 32.

A study cited in the review discovered premature sexual characteristics developed in African American girls as young as 14 months of age following topical application of hair products containing hormonally active ingredients, and regression of these characteristics occurred when product use was discontinued, although the development could persist 33. Similarly, a study of 300 women in New York found that compared to other groups, African Americans were more likely to use hair products before age 13 and reached menarche earlier; further, the use of hair oil and perm was particularly associated with earlier menarche 34. The associations described by these studies between either signs of precocious puberty or earlier menarche, and topical application of products containing EDCs, are concerning because women who are younger at menarche are at increased risk of breast cancer 10.

While the evidence for this mechanism theoretically exists, epidemiological data present with mixed findings. Data analyzed from Boston University's Black Women's Health Study, a large longitudinal cohort study, did not uncover an increased risk of breast cancer associated with hair relaxer use frequency, duration, age at first use, number of burns experienced during use, or type of relaxer used 31, although researchers did find a link between hair relaxer use and risk of uterine leiomyomata 35, another endocrine‐related disorder. However, hair relaxers are washed out relatively quickly after they are applied, whereas other products, such as leave‐in conditioner or some root stimulators, are left on the hair and scalp for longer periods of time. In another study, researchers discovered that a particular brand of root stimulator containing estrogen was significantly associated with higher mammographic density scores 36, a strong risk factor for developing breast cancer. Notably, a second study found that African Americans were the only ethnic group to use root stimulators daily 37. In this analysis, researchers discovered the strongest associations between ethnicity and product use with root stimulator, hair oil, and no lye perm/relaxer; in addition, 69% of the products selected for evaluation had placenta or a paraben listed on their labels, and more African Americans (49.4%) and African‐Caribbeans (26.4%) used these products compared to Whites (7.7%), making African American women more likely to be exposed to hormonally active chemicals in hair products 37.

Going beyond analyzing single hazardous ingredients to evaluate mixtures, another study assessed the estrogenic activity (EA) and anti‐estrogenic activity (AEA) of eight hair and skin products commonly used among African Americans, using a cell proliferation assay with an MCF‐7 cell line derived from human breast cancer 38. While findings are particular to the products tested and cannot be generalized to product type overall, of the four hair products tested, oil hair lotion demonstrated EA, conditioner with placenta and conditioner with tea‐tree oil exhibited AEA, and relaxer exhibited neither.

Although only a small number of studies have been conducted specifically exploring the role of hair products containing EDCs on breast cancer risk in African American women, they indicate a connection exists, making this an actively growing area of research (Studies reviewed are summarized in Table 2).

**Table 2:** Overview of studies investigating the relationship between hair product use and potential breast cancer risk in African American (AA) women

References	Study	Findings
Tiwary (1998) 33	Clinic patient reports of 4 AA girls (ages 14–93 months); comparison to case controls	Premature sexual characteristics developed in patients following topical application of hair products containing hormonally active ingredients, and regression of these characteristics occurred when product use was discontinued
Donovan, et al. (2007) 32	Review of 10 reports of premature or abnormal sexual development related to use of hormone‐containing products	The use of hormone‐containing products by AA girls and women helps explain the higher incidence of breast cancer in younger AAs. Early and cumulative lifetime exposure to these products may contribute to the increased lethality of the disease in this population
Rosenberg, et al. (2007) 31	Longitudinal study of 48,167 AA women including baseline and biennial follow‐up questionnaires	No increased risk of breast cancer associated with hair relaxer use frequency, duration, age at first use, number of burns experienced during use, or type of relaxer used
James‐Todd, et al. (2009) 36 *(abstract)*	Questionnaires and participant‐provided mammograms of 99 female participants of the National Collaborative Perinatal Project	One hair product, a particular brand of root stimulator, was significantly associated with higher mammographic density scores, although general hair products were not
James‐Todd, et al. (2011) 34	Cross‐sectional pilot study consisting of in‐person interviews from a convenience sample of 300 AA, African‐Caribbean, Hispanic, and White women in New York, ages 18–77	Compared to other groups, AAs more likely to use hair products before age 13 and reach menarche earlier. Use of hair oil and perm, but not other products, associated with earlier menarche
James‐Todd, et al. (2012) 37	In‐person interviews from a convenience sample of 301 AA, African‐Caribbean, Hispanic, and White women in New York; assessment of product labels for EDC content	Compared to Whites, AAs and African‐Caribbeans used more products containing EDCs or placenta, and more likely to use all types of hair products. Strongest associations between ethnicity and product use with root stimulator, hair oil, and no lye perm/relaxer; AAs the only group to use root stimulators daily. Placenta or paraben listed on the labels of 69% of the products selected for evaluation, and more AAs and African‐Caribbeans used these products compared to Whites
Myers, et al. (2014) 38	Cell proliferation assay to assess estrogenic activity (EA) and anti‐EA (AEA) of 4 hair care and 4 skin care products popular among AAs	With respect to the four hair products tested, oil hair lotion demonstrated EA, conditioner with placenta and conditioner with tea‐tree oil exhibited AEA, and relaxer exhibited neither

## Discussion

This review was initiated in response to community concern about African American women's risk of breast cancer and the use of hair products. We found that there is indeed a growing body of evidence that warrants further investigation. Although additional scientific research is clearly needed, the topic is already a source of concern in the lay community. With a number of advocacy groups calling for increased investigation, regulation, and communication around the issue, the academy has begun to respond with additional studies and collaborations. One such organization that serves as a bridge between the academic and lay communities is The Silent Spring Institute, whose researchers partner with community advocates to engage in research and outreach focusing on the impact of environmental factors on women's health, particularly breast cancer 39. This organization has published a number of studies in peer‐reviewed journals, conducting such studies as examining exposure to known carcinogens, endocrine disruptors, and other harmful compounds in ethnic personal care products. Similarly, the Breast Cancer Fund has published reports on evidence linking breast cancer to environmental exposures since 2002, and in the most recent edition, outlines concerns about the presence of EDCs and hormones in hair products, including those marketed specifically to women of color 40. In a study of the safety of chemicals found in African American hair salons in California, Environmental Finance Center West discovered numerous hair products with listed ingredients linked to breast cancer 41, found hair products containing hormones were linked to early puberty 42, and uncovered higher rates of cancer in salon workers 42. In Los Angeles, Black Women for Wellness has been exploring the links between health disparities and products used primarily by African American women and is in the process of collecting data in beauty salons 43. Researchers with the organization We Act and Touro College of Pharmacy discovered high prevalence and high usage of ethnic personal care products in Manhattan and are currently advocating for federal legislation to protect consumers from the harmful ingredients found 44.

### Policy implications

In fact, bills have recently been introduced in Congress to increase consumer protection: the Safe Cosmetics and Personal Care Products Act of 2013 45 was introduced in the House in March 2013 and the Personal Care Products Safety Act^46^ was introduced in the Senate in April 2015. Although the bills differ in their details, they are both designed to protect consumers and beauty professionals from unsafe cosmetics (the category in which hair products belong) and ingredients, including those that are known or suspected to be carcinogenic. Some of the proposed actions include establishing labeling requirements for cosmetics, requiring the Food and Drug Administration (FDA) to review the safety of a minimum number of cosmetic ingredients annually and institute safety standards to ensure ingredients do not cause harm, and allowing governmental agencies to issue recalls for dangerous cosmetics. In addition, companies are being called upon to submit full ingredient disclosure statements, including ingredient amounts, as well as safety data and reports of any adverse health events associated with their products. This is significant because current laws regulating labeling for cosmetics do not require fragrance or trade secret ingredients to be listed in descending order of predominance, as is required for other ingredients 47. Further, declaration of the ingredient mixture labeled as fragrance is currently not required 47. Another aspect of this legislation involves establishing an occupational safety and health standard for cosmetics used professionally, which is important because beauty industry employees, such as hair stylists, who work with salon products on a daily basis, may be at an even greater risk of exposure to harmful chemicals in cosmetics than consumers who are treated only occasionally. Additionally, both stylists and their customers may be unaware of all of the ingredients and chemicals contained in salon products, because the Fair Packaging and Labeling Act does not apply to products used at professional establishments, and thus, ingredient declarations are not required for the packaging of products designed for professional use and not customarily sold at retail 47.

### Strengths, limitations, and future research

One of the limitations of this review on the potential relationship between hair products and breast cancer risk in African American women is that it addresses a new topic for which there is still very little research that has been published, and thus, few studies were available to include in this review. As a result, a statistical meta‐analysis of findings could not be conducted, and instead, a mix of clinical, laboratory, and epidemiological studies were presented. While this is a limitation and can make interpretation of findings more difficult, it is also a strength in that it points to the concern that researchers from multiple disciplines have for the issue, and their varied approaches to discovering more about potential risk. Further, as reflected in the lay literature, there is clear concern from the community about this topic, although it can be difficult to bridge the lay and academic communities. As such, writing a review that both is accessible to multiple audiences and draws upon a varied set of literature, requires including quite a bit of background information, which may be considered a limitation. While one of the strengths of this study is that it describes the concern of the lay community on this topic, future research must also seek to better understand and address the challenges in communicating evolving findings to the African American public for which hair is more than just an afterthought or a potential area of risk. Hair is a complex issue in the African American community, often viewed as representing identity, race, class, heritage, and other factors 48, frequently placed at the forefront of discussions around beauty. Because hair is associated with cultural issues that are embedded and often unspoken 49, situated within a multiplicity of factors that affect individual behavior surrounding the use of hair care products, exploration of the potential causal relationship between such products and breast cancer has additional complexity.

There are many ways in which future research on breast cancer risk and hair product use should proceed and is indeed proceeding, including more sophisticated epidemiological investigations and increasing work in laboratory studies with hair products and breast cancer cells. Such work could benefit from policies that require full disclosure of ingredients and chemicals in hair products, so that researchers can describe risks more completely. In addition, while exposure to a single EDC can cause harm, contact with multiple EDCs may have a damaging additive effect, and it is important for researchers to assess “the environmental reality of long‐term low‐dose exposure to complex mixtures” 50 (p. 203) in order to better account for different compounds and concentrations, and the effects of interactions between chemicals and endogenous hormones. After more studies on this topic have been published, future reviews can overcome the challenges discussed above by focusing on the findings from particular types of research or disciplines and conducting meta‐analyses.

Through strategic partnerships, the academic, lay, and policy‐making communities have the opportunity to work together to not only better understand the role of EDCs found in hair products and breast cancer health disparities in African American women, but also to engage in a thoughtful debate about the potential “cost of beauty,” and how we can transition to safer alternatives that would allow us to move toward the elimination of these inequities.

## Conflict of Interest

There are no conflict of interest disclosures from any authors.
